# CRITTERBASE, a science-driven data warehouse for marine biota

**DOI:** 10.1038/s41597-022-01590-1

**Published:** 2022-08-06

**Authors:** Katharina Teschke, Casper Kraan, Paul Kloss, Henrike Andresen, Jan Beermann, Dario Fiorentino, Manuela Gusky, Miriam L. S. Hansen, Rebecca Konijnenberg, Roland Koppe, Hendrik Pehlke, Dieter Piepenburg, Tawfik Sabbagh, Alexa Wrede, Thomas Brey, Jennifer Dannheim

**Affiliations:** 1 Alfred Wegener Institute, Helmholtz Centre for Polar and Marine Research, Am Handelshafen 12, 27570 Bremerhaven, Germany; 2 Helmholtz Institute for Functional Marine Biodiversity at the University of Oldenburg, Ammerländer Heerstraße 231, 23129 Oldenburg, Germany; 3 University of Kiel, Institute for Ecosystem Research, Olshausenstraße 75, 24118 Kiel, Germany; 4 Hochschule Bremerhaven, An der Karlstadt 8, 27568 Bremerhaven, Germany; 5 Helmholtz-Zentrum Hereon, Institute of Carbon Cycles, Max-Planck-Straße 1, 21502 Geesthacht, Germany; 6 University of Bremen, Bibliothekstraße 1, 28359 Bremen, Germany; 7 Present Address: Thünen Institute for Sea Fisheries, Herwigstraße 31, 27572 Bremerhaven, Germany

**Keywords:** Databases, Databases

## Abstract

Data on marine biota exist in many formats and sources, such as published literature, data repositories, and unpublished material. Due to this heterogeneity, information is difficult to find, access and combine, severely impeding its reuse for further scientific analysis and its long-term availability for future generations. To address this challenge, we present CRITTERBASE, a publicly accessible data warehouse and interactive portal that currently hosts quality-controlled and taxonomically standardized presence/absence, abundance, and biomass data for 18,644 samples and 3,664 benthic taxa (2,824 of which at species level). These samples were collected by grabs, underwater imaging or trawls in Arctic, North Sea and Antarctic regions between the years 1800 and 2014. Data were collated from literature, unpublished data, own research and online repositories. All metadata and links to primary sources are included. We envision CRITTERBASE becoming a valuable and continuously expanding tool for a wide range of usages, such as studies of spatio-temporal biodiversity patterns, impacts and risks of climate change or the evidence-based design of marine protection policies.

## Introduction

Marine ecosystems provide major functions and services, which originate from the interactions of many biota a particular community consists of. However, more often than not marine biogeographical research^1,2^ and large-scale ecosystem management plans have no access to suitable community data sets for a number of reasons: (1) with increasing water depth, samples are increasingly more difficult and expensive to collect. (2) The deeper the samples are taken, the less is known about the taxonomic provenance. (3) Sorting of samples, especially of invertebrates, is time-consuming and expensive. (4) Identifying specimens requires profound taxonomic expertise, which is generally becoming rarer in the scientific community. As a result, (5) spatial resolution and coverage of samples are often limited, prohibiting large-scale analyses^3^. Moreover, (6) merging of data sets from different sources involves time-consuming synchronization of taxonomic information. Finally, (7) many data sets are not open-access and exist only in spreadsheets and local databases. These factors severely impede the scientific analysis and reuse of such data. Biogeographical research generally relies on the use of publicly available data to study large-scale biodiversity patterns in relation to environmental change, human impacts, or to be able to take protective measures^4^. Consequently, appropriate data management tools that comply with the FAIR principles (Findable, Accessible, Interoperable, Reusable) are needed to achieve an effective data and knowledge transfer to support scientific advice to decision-makers and stakeholders.

We introduce the open-access data-warehouse CRITTERBASE (https://critterbase.awi.de) for marine biota that intends to remedy these issues and facilitate functional biogeographic studies and ecosystem management approaches at multiple spatial scales. The development and implementation of CRITTERBASE focused on benthic data so far, because this is our main field of expertise and here we see the most urgent need to act.

Benthic communities play a key role in processes of important functions and services of marine ecosystems^5,6^. They contribute significantly to overall marine biodiversity, constitute important parts of marine food webs, and facilitate nutrient recycling at the sediment-water interface^7^. Moreover, benthic organisms are excellent sentinels of changing environmental conditions^8^, such as eutrophication^9^, owing to the close association between environmental drivers and benthic distribution patterns^10,11^ and to the comparatively long lifespans of benthic organisms. Benthic biodiversity data that cover large spatial scales and long temporal scales at high taxonomic resolution are therefore of pivotal importance for marine ecosystem management and environmental protection to ensure a sustainable use of coastal and offshore systems. Key examples of this are management approaches set out in national and international guidelines, such as the Marine Strategy Framework Directive (MSFD) of the European Union, or marine spatial planning focusing on the key functions of ecosystems^12^.

## Results

### Application state

**Fig. 1:**
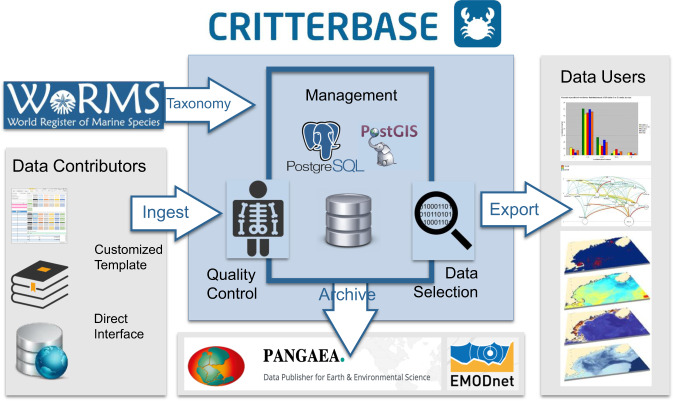
Workflow of CRITTERBASE. Ingesting data into the data-warehouse and generating output for data visualization and extraction.

**Fig. 2:**
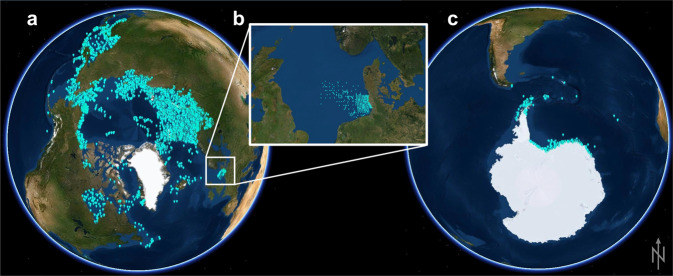
Map of 14,212 sampling stations in (**A**) Arctic seas (11,561 stations), (**B**) the North Sea (2,119 stations), and (**C**) the Weddell Sea and adjacent seas (Antarctica) (532 stations).

Currently, CRITTERBASE hosts data on benthos from Arctic, North Sea, and Antarctic regions (see Table 1), as these are the geographic foci of our research at the Alfred Wegener Institute (AWI). CRITTERBASE uses a shared data model for data from all geographic regions that safeguards the integrity of data regardless of whether they are collated from literature^13^, cooperating researchers, own research, archives or repositories^14^. To this end, it utilizes a single standardized workflow (Fig. 1). Only data with metadata on sampling location and date, taxonomic resolution, and sampling method were included, leading to the spatial extent of current records shown in Fig. 2. Data quality controls are major components within CRITTERBASE and ensure that the imported data meet a high quality standard. There are basic quality components, such as its data model itself, and several other routines that flag mistakes through a number of logical checks before, during and after data import to prevent data errors that may corrupt subsequent analyses. Further details on the quality control components are provided at https://critterbase.awi.de/#qc.

**Table 1:** Summary of CRITTERBASE contents.

Source	Area	Period	*n*-samples	Sampling method	Taxonomic resolution
PANABIO	Arctic seas	1800–2014	11,575	Photographs, various trawling and grab methods	Species, genus
BENOSIS	North Sea	1969–2007	6,435	Various grabs, box cores, otter trawls and beam trawls	Species
WEECOS	Weddell Sea	1983–2004	634	Various grabs, trawls, traps and photographs	Species to phylum

### Data provenance

The marine benthic data collated originated from three regional projects: PANABIO - PAN-Arctic Information System of Benthic BIOta (Arctic), BENOSIS - Benthic North Sea Information System (North Sea), and WEECOS - WEddell Sea Integrated ECOSystem (Antarctic).

The majority of the Arctic data originated from a pan-Arctic inventory of Arctic benthic fauna^13^ as part of the Census of Marine Life’s Arctic Ocean Diversity project, or had been published by the data publisher PANGAEA (www.pangaea.de), e.g. Andrade *et al*.^14^. Most records were identified to species level, but some to genus level only. Users can find trait information of Arctic benthos in the open-access Arctic Traits Database^15^, which, like CRITTERBASE, also uses the World Register of Marine Species (WoRMS; www.marinespecies.org) to assign taxonomic identities.

The North Sea benthic data are a synthesis of 13 projects. Data include grab and trawl samples taken in the southeastern North Sea between 1969 and 2007. The organisms have been identified to the lowest taxonomic level possible, i.e. the majority of data are available at species level. Data from four projects have been published^16–19^, while the remaining nine data sets will be made available in CRITTERBASE in due course.

Antarctic benthic data^20–24^ were compiled, processed and uploaded to PANGAEA as part of the Weddell Sea marine protected area planning project^25,26^, with the exception of data already published by Gerdes *et al*.^27–41^. The data mainly include trawls and grab samples taken in the Weddell Sea and adjacent seas between the early 1980s and mid-2000s (Table 1). Most data are at species level and have a particular focus on echinoderm taxa. Two data collections encompass only higher taxonomic groups (mostly at the level of order or higher).

### Data query

CRITTERBASE is an open-access information system, allowing users to query and download data as Excel files for further processing. Users are kindly asked to cite this paper in any publication or report and provide attribution to the original data sources that form the basis of CRITTERBASE. CRITTERBASE’s query functions allow for the selection of data records based on (1) location (structured according to the regions used by the Food and Agriculture Organization of the United Nations, FAO), (2) sampling gear (e.g., trawl, grab, video) and (3) data set. Each data record is stored as part of a unique data set, which is accessible via a unique identifier (DOI or reference to the original publication containing the data) and generally represents a cruise or a research project. Data records represent the occurrence of a specific taxon at a given geographic position at a given time. Additional information on the abundance, biomass and life stage of the taxa are provided for 25%, 20% and 0.6% of all data sets respectively. Each data record has a unique identification number within CRITTERBASE. Taxonomic information is recorded using AphiaIDs of WoRMS that allow for easy scaling from species to higher taxonomic level.

## Discussion

The main objective of CRITTERBASE is to promote the sharing of and easy access to data for the marine biology community, serving as a tool for collaboration while also safeguarding valuable data for future use. The data can be used, for example: (*i*) to support impact studies (e.g., effects of trawling from commercial fishing or the influence of offshore wind farms), (*ii*) as a knowledge base to be used for marine spatial planning and associated management and monitoring activities (e.g., within the framework of the MSFD, the EU Habitats Directive, or the International Council for the Exploration of the Sea [ICES]), *(iii)* to assess trends of benthic biodiversity over space and time^42^, such as species’ range shifts or the introduction of invasive species due to climate change^43–45^.

CRITTERBASE provides a valuable resource for research in polar and temperate regions, as well as for scientists aiming to address large-scale patterns of biodiversity. However, there are a few caveats that need to be considered due to the large variety of sampling methods and taxonomic resolution. Benthos samples were collected on board of research vessels with commonly used methods, such as trawling, grab sampling, or recorded via seabed imaging by means of ocean-floor observation systems and variations thereof^13^. The sampling methods cover different, yet overlapping, parts of the benthic communities. Grab samples primarily contain macrobenthic fauna, trawl catches encompass megabenthos, and seabed images give information on epibenthos. This difference in species-scope is noted in the associated metadata and should be considered when querying or analyzing data. Sampling depths range from coastal waters to areas on the continental shelf and the deep sea (specifically, from a few meters to more than 4000 m). Samples were processed using standard operating procedures, such as the ICES 1999 ISO standard for grab sampling. However, it should be noted that this was not done homogeneously across areas and is not standardized across data sets. Metadata detail each entry’s methodological variations, and, where available, a reference is provided for each data set to provide more detail on the applied methodologies and their limitations. For example, some research cruises were purely focused on certain taxonomic groups^46^, thus restricting the taxonomic scope of the data. This is also noted in the metadata and should be carefully reviewed while querying or processing the data. Taxonomic adjustments of the data might be necessary depending on the user’s needs. For instance, it may be necessary to aggregate species-level taxa to higher taxonomic groups when merging data sets with different taxonomic resolution. It should also be noted that the records currently within CRITTERBASE indicate only the presence of a recorded taxon. While absences can be inferred from the taxonomic scope of each data set, this is a decision that has to be made by the data analyst and is case-dependent.

In summary, CRITTERBASE’s extensive metadata (from information on catchability, such as mesh size or trawling speed, to details on the taxonomic resolution and coverage) provides the best possible transparency of the data. This, in turn, allows users to perform analyses on joint data sets and thereby contributes to a better understanding across large temporal and spatial scales. It is important to note, however, that case-by-case decisions are necessary to appropriately pool or aggregate the partly inconsistent data sets (i.e., data sets differing in sampling approaches) provided by CRITTERBASE so that the data can eventually be used for joint analyses^47^. Data analysts should pay adequate attention and be vigilant in order to correctly merge the data for their purpose before starting any data analysis. Negligent use of the data provided by CRITTERBASE could lead to wrong ecological conclusions regarding, for instance, temporal and spatial trends of biodiversity.

Future releases of CRITTERBASE will contain more software features and will include data on more diverse biota. We aim to maintain CRITTERBASE as a common open access platform. We plan to grow the platform by including data from other researchers or groups (e.g. ICES WGMBRED data initiative). While the current version of CRITTERBASE includes benthic invertebrates only, future developments of the data model will allow for the ingestion of data from other marine realms, such as pelagic and under-ice invertebrates and fish. CRITTERBASE will also be upgraded to handle information on functional traits and further organism-specific properties^48^. This will then encourage and facilitate trait-based approaches (alongside species-based methods) to, for example, improve our understanding of the processes that influence biodiversity-ecosystem functioning^9^ and to assess trends in ecosystem functioning over space and time. In addition, we will extend the CRITTERBASE platform with routines (implemented in R or Python) for the calculation of metrics useful for further applications, such as estimating secondary production or carrying out effective survey planning.

## Methods

### Data compilation

The compilation of data included in this release of CRITTERBASE consisted of two initial steps. First, we defined a data model for CRITTERBASE that would serve Arctic, North Sea, and Antarctic data demands for current and future use (for details on the data model, see https://critterbase.awi.de/#qc#critterbase-data-model).

For each geographic area, efforts had already been made to build a data-warehouse that would support species distribution modeling (e.g., PANABIO^13^ in the Arctic seas), human-impact studies (e.g., benthos database for ecological research in the North Sea: BENOSIS), or marine conservation planning in the Weddell Sea^25,26^ (WEECOS). These previous efforts were merged into a general data model through iterative discussions with the data warehouse developers. We then ingested already available data into CRITTERBASE (Table 1), including those available from PANGAEA^14^ and our own research. The data were then quality checked by AWI experts in data collection and taxonomic identification of benthic communities.

As a result of the data compilation process, CRITTERBASE’s initial data sets reflect the research needs of the department of Functional Ecology within AWI. They represent a first stock of benthic data and serve to demonstrate and develop the benefits of using CRITTERBASE when compared to traditional spreadsheet-based archives. The open-access CRITTERBASE web interface allows any user to query and download data for further processing for any purpose.

### Data quality control

**Fig. 3:**
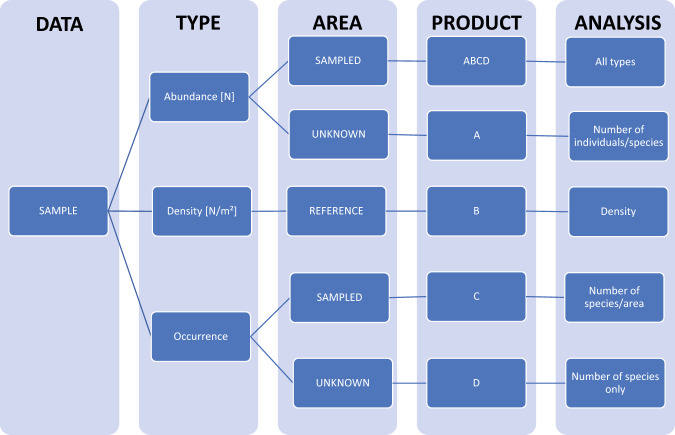
Flow chart indicating the various data scenarios for biota and sampling area input in CRITTERBASE. Starting from taking a sample, biotic data can be abundances, densities (i.e. in our case individuals/m²) or occurrences. Depending on the availability of sampling area, i.e. actually sampled area, reference area (fictional/defined area; here: 1 m^2^) or unknown area, possible data product types are ABCD, A, B, C, or D. A allows to assess the relationship between number of individuals and number of species per sample, B allows for the assessment of density, C only allows for the determination of the relationship between number of species and area, whereas D only allows for the determination of the number of species per sample. Type ABCD supports all types of biodiversity analysis. An incorrect pairing of Type and Area (e.g., abundance with reference area) produces an error message upon attempted import into CRITTERBASE. If there is actually no information on the sampled area, unknown area must be selected (which is accepted and leads to Product A). If information on the reference area (instead of sampled area) is given, abundance values would have to be entered as densities. This quality check ensures that the data type and area are consistent.

The automated quality management procedures built into the CRITTERBASE Collector App - the backend of the CRITTERBASE web interface - prevent the import of incorrect or incomplete data sets. Common mistakes are identified through a number of logical controls before, during and after data import. These include, for instance, the detection of differing sampling dates, coordinates or water depths within a single sample. In addition, any new taxonomic name not previously imported via a data set is validated against the current taxonomic classification provided by WoRMS. This prevents the use of synonyms, any incorrect spelling of scientific names, or the use of outdated names, all of which potentially inflate biodiversity estimates and skews species distributions. We also implemented checkpoints in the CRITTERBASE Collector App to verify the type of data we are dealing with (e.g., presence/absence versus abundance data), which ultimately decides on the kinds of biodiversity analysis possible (Fig. 3). Two types of error messages are possible during data ingestion: a complete rejection of the data due to critical errors that comprise the integrity of the database and need to be solved immediately, or minor warnings indicating mistakes that could be improved to increase the data quality, such as small differences in the spelling of scientific names (see details on CRITTERBASE quality management and its control components at https://critterbase.awi.de/#qc).

In addition, the CRITTERBASE Collector App, available at Zenodo (10.5281/zenodo.5724021^49^), enables users to create their own CRITTERBASE to manage ecological data projects on their own devices. It sets up a clean PostgreSQL object-relational database with the spatial database extender PostGIS and allows users to quality-check and store biological data (using the CRITTERBASE data model) without having to share their data through the open-access CRITTERBASE web interface (https://critterbase.awi.de). Users working locally can make direct queries to their CRITTERBASE via SQL, R and Python, allowing users to keep data queries and code neatly in one place for subsequent analyses. We hope that the option for decentralized working with the CRITTERBASE Collector App - as an open-source tool - will make this an appealing data management option for other researchers, resulting in more processed and quality-checked data sets, which in turn could be made available for publication via the CRITTERBASE web interface.

## Data Availability

All data from Arctic, North Sea, and Antarctic regions, discussed in this paper, are publicly available at https://critterbase.awi.de. Continuous online access to CRITTERBASE will be ensured through a permanent data portal hosted by the Alfred Wegener Institute, Helmholtz Centre for Polar and Marine Research (https://marine-data.org). New data will be published as they are submitted and meet the quality standards.

## Code availability

The source code of the web service and portal (CRITTERBASE Version 1.0.0) - for accessing quality-checked data sets - is released under the BSD 3 license and is publicly available via the following stable link: https://critterbase.awi.de/code. The software libraries and versions used are referenced in the source code.

The Collector App for CRITTERBASE - the software for data quality checking and storage - is available as a free download under the open-source BSD 3 license (10.5281/zenodo.5724021^49^). The software libraries and versions used are referenced in the README.MD file.
